# A Review of Intraoperative Goal-Directed Therapy Using Arterial Waveform Analysis for Assessment of Cardiac Output

**DOI:** 10.1155/2014/702964

**Published:** 2014-05-27

**Authors:** Neil Mehta, Ana Fernandez-Bustamante, Tamas Seres

**Affiliations:** Department of Anesthesiology, University of Colorado School of Medicine, 12405 East 17th Avenue, Aurora, CO 80045, USA

## Abstract

Increasing evidence shows that goal-directed hemodynamic management can improve outcomes in surgical and intensive care settings. Arterial waveform analysis is one of the different techniques used for guiding goal-directed therapy. Multiple proprietary systems have developed algorithms for obtaining cardiac output from an arterial waveform, including the FloTrac, LiDCO, and PiCCO systems. These systems vary in terms of how they analyze the arterial pressure waveform as well as their requirements for invasive line placement and calibration. Although small-scale clinical trials using these monitors show promising data, large-scale multicenter trials are still needed to better determine how intraoperative goal-directed therapy with arterial waveform analysis can improve patient outcomes. This review provides a comparative analysis of the different arterial waveform monitors for intraoperative goal-directed therapy.

## 1. Introduction

There is increasing evidence that intraoperative fluid and hemodynamic management influence patient outcomes. It is a challenge for anesthesiologists to balance between administering intravenous fluid, vasoactive agents, or inotropic drugs to maintain appropriate cardiac output. Individualized goal-directed therapy (IGDT) utilizes hemodynamic parameters such as stroke volume, cardiac output, cardiac index, peripheral vascular resistance, blood pressure, and the variation of stroke volume to optimize volume status, myocardial contractility, and tissue perfusion. Previous studies have demonstrated that IGDT in the perioperative period can improve patient outcomes by decreasing postoperative recovery time, reducing postoperative complications, and shortening hospital length of stay, particularly in high-risk surgical patients [1–19].

Different monitoring techniques are available to evaluate stroke volume and cardiac output for IGDT intraoperatively. Since cardiac output is the principal determinant of tissue oxygen delivery, any monitoring technique used to guide fluid therapy should measure cardiac output [20]. One such technique is arterial waveform analysis, which evaluates stroke volume to calculate cardiac output and examines stroke volume variation to assess fluid responsiveness. While other intraoperative cardiac output monitors are available, such as pulmonary artery thermodilution and esophageal Doppler echocardiography, this review will focus on the use of intraoperative arterial waveform analysis for IGDT.

## 2. Methods

### 2.1. Basic Concepts of Arterial Waveform Analysis

Arterial waveform analysis is based on the relationship between blood pressure, stroke volume, arterial compliance, and vascular resistance. Different models and methods are used for the mathematical analysis of this waveform, one of them is the Windkessel model. This model originated from the Windkessel effect described in a circuit where there is an air chamber between a hand-operated pulsatile water pump and a water tube. As water is pumped periodically into the circuit, it compresses the air in the chamber which, in turn, pushes the water out of the chamber and into the circuit. The air chamber dampens the fluctuation of the water flow. The Windkessel effect can be observed in the human circulatory system when large elastic arteries distend as the blood pressure rises during systole and recoil as the pressure falls during diastole. The Windkessel effect dampens the fluctuation of blood pressure during the cardiac cycle and maintains organ perfusion during diastole. This model in human circulation is based upon two assumptions. The first is the conservation of mass principle, which states that the flow into a blood vessel must be equal to the outflow. The second assumption is that the compliance of the vessel affects its flow. During systole, the pressure in the blood vessel causes expansion and absorbs some blood because of the peripheral vascular resistance. During diastole, the pressure decreases and the stored blood is expelled. The peripheral vascular resistance and the capacitance of the arteries to store blood are the basis of the 2-element Windkessel model [21].

**Figure 1:**
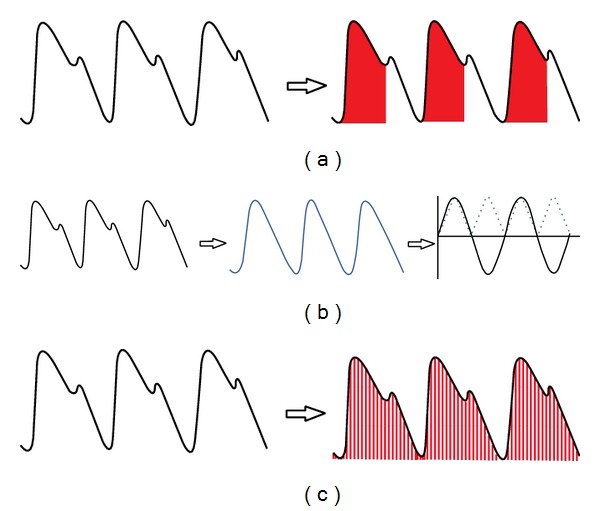
Different methods of arterial waveform analysis. (a) The PiCCO system utilizes the area under the curve of the systolic portion of the arterial waveform to calculate cardiac output, as depicted by the shaded area of the waveform on the right. (b) The LiDCO system uses pulse power analysis by first transforming the arterial waveform into a volume versus time waveform, as shown in the middle of the panel. Next, autocorrelation using a sine wave (solid black curve on the right of the panel) and a sine squared wave (dotted blue line on the right of the panel) estimates a nominal stroke volume, which can then be converted to cardiac output via calibration. (c) The FloTrac system samples multiple data points continuously, as depicted by the red lines. The standard deviation of the pressure data points around the mean arterial pressure is linearly related to stroke volume, which is then used to calculate cardiac output.

The 2-element Windkessel model is used for pulse contour analysis in the PiCCO (Pulsion Medical Systems, Munich, Germany) system. The cardiac output and aortic compliance are obtained by transpulmonary thermodilution via a central line. Once calibrated, the area under the systolic portion of the arterial pressure waveform is calculated on a beat-to-beat basis (Figure 1(a)). Because of the change in peripheral vascular resistance during monitoring, the system needs periodic recalibration for accurate stroke volume measurement [20, 22, 23].

The algorithm applied in the LiDCO (LiDCO, London, United Kingdom) system uses pulse power analysis rather than the shape of the arterial waveform. It is based on the assumption that the net power change in a heartbeat is dependent on the balance between the input of a mass of blood from stroke volume minus the blood lost to the periphery. Based on the conservation of mass and the correction for compliance, there is a linear relationship between net power and net flow. In this technique, the arterial pressure waveform is first converted into a volume waveform and then an autocorrelation technique is applied. This autocorrelation utilizes a continuous sine wave to describe the periodic motion of blood during a cardiac cycle and the root mean square of the sine waveform to describe a nominal stroke volume (Figure 1(b)). This volume is then converted to the actual stroke volume either by calibration or via comparison to a database as described below [20, 22, 23]. In the LiDCO device, a transfer function is used to relate peripheral pressures to central pressures. Because the peripheral pressure is measured directly, central pressure can be estimated with either a mathematical model or population data [24, 25].

The FloTrac (Edwards Lifesciences, Irvine, California) system provides an estimate of cardiac output using the standard deviation of the arterial pulse pressure around the mean arterial pressure and a conversion factor. The system samples data points at 100 Hz for 20 seconds to calculate the standard deviation of the pulse pressure. The conversion factor represents systemic vascular resistance, arterial compliance, body surface area, and biometric modifiers obtained from demographic data (Figure 1(c)). This algorithm does not require calibration because the conversion factor autocorrects for changing peripheral vascular resistance [20].

There is an additional method that utilizes pulse contour analysis and does not require preloaded data or calibration. It is called the MostCare (Vytech, Padova, Italy) system but because there are fewer validation studies with this system and no intraoperative trials, it is not included in this review [22].

### 2.2. Systems for Arterial Waveform Analysis

Four different systems are available to analyze the arterial waveform for monitoring cardiac output and guiding fluid therapy: FloTrac, LiDCO, PiCCO, and MostCare. As explained above, the MostCare system is not described in further detail in this review. The other three systems have been validated using comparisons to gold standard techniques for cardiac output monitoring, most commonly thermodilution with a pulmonary artery catheter. The systems are different in terms of invasiveness, calibration, and limitations (Table 1). These three systems have been used in randomized controlled trials to assess IGDT intraoperatively and its effect on patient outcomes. One important limitation of all the systems is that they do not perform reliably in hemodynamic instability, although the LiDCO and PiCCO systems may perform better in these situations [20].

#### 2.2.1. The FloTrac System (Edwards Lifesciences, Irvine, California)

The FloTrac system involves a FloTrac sensor and a Vigileo monitor and is also known as FloTrac/Vigileo. It requires a peripheral arterial line and it does not require calibration. There are currently three different software releases of the system. The software updates have improved the validity and reliability of the measurements. However, it is still unclear how reliable the system is in low systemic vascular resistance states, such as in patients with sepsis or in patients who are on concurrent vasopressor therapy [20, 24, 26, 27].

#### 2.2.2. The LiDCO System (LiDCO, London, UK)

The LiDCO system requires an arterial line and a calibration system using a lithium indicator dilution. With the new LiDCO plus system, recalibration is not necessary. Additionally, a LiDCO rapid version that consists of the previously described pulse power analysis algorithm and does not require calibration at all exists. The LiDCO rapid system is able to do this by the use of patient biometric data, including age, height, and weight, which serve as the calibration for the system [28]. One important limitation for the LiDCO systems requiring calibration is that, in patients receiving lithium therapy, the baseline lithium level will falsely elevate the calculated cardiac output. Moreover, neuromuscular blockers that have quaternary ammonium ions can disturb the lithium sensor and affect estimated cardiac output. Nonetheless, the LiDCO method has still been shown to be at least as reliable as other thermodilution techniques, and it also only requires a peripheral arterial line. Furthermore, because LiDCO does not use pulse contour analysis but rather employs pulse power analysis, the shape of the waveform is not as important. Additionally, the LiDCO system may be accurate in cases of hemodynamic instability although data are still inconclusive [20, 24, 26, 29].

#### 2.2.3. The PiCCO System (Pulsion Medical Systems, Munich, Germany)

The PiCCO system combines arterial waveform analysis with thermodilution techniques. It uses transpulmonary thermodilution, which requires both central venous and central arterial access (femoral, axillary, or brachial artery). External measurement of the cardiac output and the compliance of the aorta via thermodilution provide the calibration factor. The PiCCO system has been shown to be reliable when compared with a pulmonary artery catheter in a variety of situations and may even have good tracking of cardiac output in cases of hemodynamic instability, although data are still inconclusive. Additional benefits include more calculated data, such as extravascular lung water or intrathoracic blood volume. The major drawback of the PiCCO system is the requirement of central arterial and venous access as opposed to the LiDCO and FloTrac systems which require only peripheral arterial access. Additionally, there is no data to describe how often recalibration is needed [20, 24, 26, 39].

### 2.3. Article Search

This paper is an unsolicited review to determine if intraoperative IGDT applied by different systems using arterial waveform analysis improves patient outcomes and when this technique should be employed. To gather appropriate articles about trials of intraoperative IGDT, a PubMed search was undertaken using search phrases coupling “goal-directed therapy” with “arterial waveform analysis” and with each of the three systems. This search provided forty articles. Articles that were randomized controlled trials were included for further analysis and the rest were excluded. This yielded a total of three articles for this review. In an effort to find more literature, all review articles from the original search were read and citations for further articles that were randomized controlled trials involving any of the three systems were reviewed. This technique gave an additional six articles for review, providing a total of nine trials.

## 3. Results

### 3.1. Trials of Intraoperative IGDT Using Arterial Waveform Analysis

Nine small-scale randomized controlled trials have been undertaken to examine patient outcomes when performing goal-directed therapy using arterial waveform analysis intraoperatively (Table 2). To date, no large-scale multicenter trials have been done.

#### 3.1.1. FloTrac Trials

Benes et al. completed a small prospective randomized trial in high-risk surgical patients using the FloTrac system to optimize intraoperative fluid management. There were 60 patients in both the control and FloTrac groups, all of whom were scheduled for elective intra-abdominal surgery. The aim of this study was to maintain stroke volume variation less than 10% with colloid boluses of 3 mL/kg. Patients in the FloTrac arm had significantly fewer hypotensive events intraoperatively, lower lactate levels at the end of surgery, and fewer postoperative complications (18 versus 35 patients in the FloTrac and control groups, resp.). Severe complications (7 versus 22 patients in the FloTrac and control groups, resp.) and total complications (34 versus 77 patients in the FloTrac and control groups, resp.) were also significantly decreased. No difference in hospital length of stay or mortality was seen [30].

Mayer et al. had similar results in a separate small randomized controlled trial of high-risk surgical patients. This study had a total of 60 patients, 30 in each group, all scheduled for major abdominal surgery. The FloTrac system was used to maintain a cardiac index greater than 2.5 L/min/m^2^ using either dobutamine or colloid boluses, depending on stroke volume index and stroke volume variation. In this study, significantly fewer patients developed complications in the FloTrac arm (6 versus 15). There was a significantly shorter median duration of hospital stay (15 days versus 19 days) in the FloTrac group compared to the control group [31].

Cecconi et al. performed a randomized controlled trial of goal-directed therapy using the FloTrac system in patients undergoing elective total hip arthroplasty under regional anesthesia. This small-scale study included 20 patients each in the control arm and the FloTrac arm. In the FloTrac group, patients received colloid boluses until stroke volume increases were less than 10%. At that time, if oxygen delivery was not greater than 600 mL/min/m^2^, dobutamine was started and increased to reach the oxygen delivery goal. Blood samples were taken every 30 minutes and hemoglobin concentration was maintained greater than 10 g/dL. Goal-directed therapy applied to these patients showed statistically significant decreases in postoperative complications in the FloTrac arm, although the number of complications was small in both groups. Patients in the FloTrac arm received more blood intraoperatively; however, the control group needed more transfusions postoperatively. Overall, the quantity of blood transfused was the same between the groups. The FloTrac arm did receive more dobutamine intraoperatively (11 of 20 patients versus 0 in the control arm) [32].

Scheeren et al. conducted a prospective, randomized multicenter study of high-risk surgical patients to evaluate FloTrac based intraoperative goal-directed therapy. The treatment group had stroke volume variation maintained at less than 10% with colloid boluses. The study included 64 patients undergoing high-risk surgery, with 32 patients enrolled in each arm. Postoperative wound infections were lower in the FloTrac group and this data reached statistical significance (0 patients versus 7 patients in the control group). There was a trend toward fewer complications in the FloTrac group, although this was not statistically significant in this study. Additionally, intensive care unit (ICU) length of stay tended to be shorter in the FloTrac group, but this was also not statistically significant [33].

Finally, van der Linden et al. performed a randomized controlled trial to evaluate the effectiveness of goal-directed therapy with the FloTrac system in patients undergoing peripheral arterial surgery. The main outcome measure for this study was tissue oxygen delivery. Cardiac index was to be maintained greater than 2.5 L/min/m^2^ using colloid boluses initially and as long as cardiac index increased, this was maintained until central venous pressure was 15 mm Hg at which time dobutamine was initiated. The study had 3 different groups: group 1 underwent standard hemodynamic management with sevoflurane based general anesthesia, group 2 received goal-directed therapy with sevoflurane based general anesthesia, and group 3 was administered goal-directed therapy with propofol based general anesthesia. Patients assigned to goal-directed therapy with the FloTrac system received more dobutamine intraoperatively (2 patients in group 1, 13 patients in group 2, and 12 patients in group 3). None of the patients in the sevoflurane groups had postoperative cardiac complications but 4 of 20 patients in the propofol group had postoperative cardiac complications. In terms of tissue oxygen delivery, no differences between any of the groups were seen [34].

#### 3.1.2. LiDCO Trials

There are a limited number of trials utilizing the LiDCO system for intraoperative goal-directed therapy. Pearse et al. conducted a randomized controlled trial for early goal-directed therapy using the LiDCO system following major surgery. There were 122 patients in this study, 62 patients in the treatment arm, and 60 patients in the control group. The goal of the treatment arm was to attain an oxygen delivery index of 600 mL/min/m^2^ versus conventional management in the control group. The treatment group received more colloid and dopexamine to maintain oxygen delivery. Statistically significant findings included a reduction in complications and median duration of hospital stay. No difference in mortality was seen. Because this is a postoperative study, it is not included in Table 2 [40].

Bisgaard et al. performed a randomized controlled trial using LiDCO based goal-directed therapy in the perioperative period in patients undergoing open abdominal aortic surgery. 64 patients were enrolled in the study (32 in each group). LiDCO data was used prior to surgery and continued until 6 hours postoperatively. Stroke volume index was monitored and boluses of 250 mL of colloid were given in the LiDCO group to maintain stroke volume index intraoperatively. Postoperatively, colloid boluses were given and dobutamine was initiated if oxygen delivery did not reach 600 mL/min/m^2^ after stroke volume index optimization. Stroke volume index and oxygen delivery index were higher in the postoperative period in the IGDT group; however, the number of complications and length of hospital stay did not differ between the groups [35].

A different study by Bisgaard et al. evaluated the use of goal-directed therapy in patients receiving lower limb arterial surgery. This study was also conducted from the start of surgery to 6 hours postoperatively. This study had 40 total patients with 20 patients each in the LiDCO group and the control group. The protocol in this study is the same as above. Boluses of 250 mL of colloid were given in the LiDCO group to maintain stroke volume index intraoperatively. Postoperatively, colloid boluses were given and dobutamine was initiated if oxygen delivery did not reach 600 mL/min/m^2^ after stroke volume index optimization. Stroke volume index and cardiac index throughout the treatment period and postoperative oxygen delivery were improved for patients in the LiDCO group. Complications were significantly lower in the LiDCO group (5 of 20 patients) versus the control group (11 of 20 patients). There was no difference in the median length of hospital stay between the groups [36].

In addition to these studies, there is an additional study that is currently underway examining goal-directed therapy intraoperatively with the LiDCO system. Wiles et al. have proposed this study to look at patients undergoing hip fracture surgery who receive spinal anesthesia. The study has been approved and is registered but no data is currently available. The abstract methods state that the plan is to enroll a total of 128 patients [37].

#### 3.1.3. PiCCO Trials

While the PiCCO system has been well validated, it has not been used in randomized clinical trials as much as the other methods of arterial waveform analysis. Goepfert et al. did utilize the PiCCO system in 100 patients undergoing coronary artery bypass grafting to determine if individualized therapy could improve outcomes. This study was started intraoperatively and continued throughout the ICU course. Goal-directed therapy focused initially on maintaining stroke volume variation below 10% by use of intravenous fluids. Then, cardiac index was maintained at 2 L/min/m^2^ either with heart rate increases via pacing if heart rate was less than 50 beats per minute or with epinephrine. Norepinephrine was given if the cardiac index was appropriate but the mean arterial pressure was less than 65 mm Hg. Statistically significant findings included patients in the treatment group (*n* = 50) having fewer postoperative complications than the control group (*n* = 50), 40 versus 63, taking less time to achieve ICU discharge criteria (15 hours versus 24 hours), and having shorter ICU stays (42 hours versus 62 hours), respectively [38].

## 4. Discussion

Many different tools now exist to help anesthesiologists measure cardiac output intraoperatively. For many years, we had relied only on data from pulmonary artery catheters. Arterial waveform analysis with FloTrac and LiDCO provides the option to use only a peripheral arterial line for cardiac output measurement. The PiCCO system offers an additional option if a central arterial line and a central venous line are placed. While no large multicenter studies exist for utilizing this new technology, small-scale studies suggest fewer complications and decreased hospital length of stay when anesthesiologists use arterial waveform analysis in the operating room to guide goal-directed therapy.

In light of recent evidence that goal-directed therapy improves patient outcomes, these early trials are not surprising but nonetheless provide an exciting new area of research. In addition to large-scale studies, parameters need to be defined to guide goal-directed therapy, including when and how much fluid to give and when to initiate inotropes. Moreover, not all patients would benefit from additional monitoring so more definition needs to be given to the specific patient populations and types of surgeries where arterial waveform analysis should be used. Additionally, more evidence is needed to decide if analysis should be done intraoperatively, postoperatively, or both.

Our experience with arterial waveform analysis for cardiac output monitoring in the intraoperative setting has included the LiDCO and FloTrac systems. A first generation LiDCO device was used to keep the cardiac index at the basal value, which was determined at the start of the case. This preliminary system did not provide stroke volume variation so the arterial waveform was monitored for significant amplitude variation. When this occurred, volume was administered to overcome pressure variation and hypotension. The first generation LiDCO system has significantly changed since that time, and we do not have experience with the current LiDCO models. We have, however, recently used the FloTrac system in major abdominal and vascular cases and monitored cardiac index and stroke volume variation to guide intraoperative fluid management.

Based on our experience, we do not currently see an advantage of one system over another. They both provide practitioners algorithms for hemodynamic management, which is the first step in optimizing the amount of fluid and vasoactive medications administered intraoperatively. Thus far, we have used various systems based primarily on availability and recommend that providers use whichever systems that are readily available and well understood. Our future steps include the implementation of prospective studies to better understand the use of arterial waveform analysis in specific patient populations, such as in patients with hypertension or decreased ejection fraction.

## 5. Conclusions

While there is a lack of large multicenter randomized controlled trials, preliminary small-scale studies indicate that utilizing intraoperative arterial waveform analysis to guide IGDT improves patient outcomes. These studies have shown fewer postoperative complications, fewer wound infections, and decreased hospital length of stay when arterial waveform analysis is used intraoperatively. The appropriate selection of a system can vary based on the patient, procedure type, and institutional variation, and more studies need to be completed to further define these parameters.

The FloTrac system seems to have the most data and is also the easiest to use for the fact that it requires only a peripheral arterial line and does not require calibration. The LiDCO system also requires only a peripheral arterial line; however, certain versions do require calibration. An added benefit is that the LiDCO system uses pulse power analysis and therefore does not rely on the shape of the arterial waveform. The PiCCO system is the most cumbersome as it requires both central arterial and central venous access as well as calibration. Studies have just begun with the PiCCO system so it is still unclear whether the benefits outweigh these disadvantages. An important aspect of this system that should be considered is that it provides additional information including extravascular lung water and intrathoracic blood volume, which can be important in critically ill patients.

## Figures and Tables

**Table 1:** Overview of the different arterial waveform analysis systems.

	FloTrac	LiDCO	PiCCO
Method of analysis	Standard deviation of arterial pulse pressure around the mean arterial pressure	Pulse power analysis	Pulse contour analysis

Calibration	Not needed	Manual—lithium dilution (not needed in LiDCO rapid)	Manual—thermodilution with saline or glucose

Requirements	Peripheral or central arterial	Peripheral or central arterial	Central arterial and central venous

Advantages	Minimally invasive, easy to use, and no calibration	Minimally invasive, easy to use, no calibration with LiDCO rapid, more accurate with hemodynamic instability, and waveform shape does not matter	More accurate with hemodynamic instability, additional data available (extravascular lung volume and intrathoracic blood volume)

Disadvantages	Not as reliable with hemodynamic instability since peripheral vascular resistance is included in the conversion factor	Not as accurate when patient receive lithium therapy or certain neuromuscular blocking agents	More invasive, shape of arterial waveform matters

**Table 2:** Studies using IGDT with arterial waveform analysis intraoperatively.

Study authors	Analysis system	Type of study	Total number of patients	Outcomes
Benes et al. [30]	FloTrac	RCT	120	FloTrac group had significantly fewer postoperative complications. No difference in hospital length of stay or mortality was seen.
Mayer et al. [31]	FloTrac	RCT	60	FloTrac group had significantly fewer complications and a shortened median duration of hospital stay.
Cecconi et al. [32]	FloTrac	RCT	40	FloTrac group had a significant decrease in postoperative complications and received more dobutamine intraoperatively.
Scheeren et al. [33]	FloTrac	RCT	64	FloTrac group had significantly fewer postoperative wound infections. No significant difference in complications or ICU length of stay.
van der Linden et al. [34]	FloTrac	RCT	27	No difference in tissue oxygen delivery (main outcome measure).
Bisgaard et al. [35]	LiDCO	RCT	64	LiDCO group had higher stroke volume index and oxygen delivery index in postoperative period. No difference in number of complications or length of hospital stay.
Bisgaard et al. [36]	LiDCO	RCT	40	LiDCO group had increased stroke volume index, cardiac index, and oxygen delivery. Statistically significant decrease in complications in LiDCO group. No difference in the median length of hospital stay.
Wiles et al. [37]	LiDCO	RCT	128 (planned)	Ongoing. No available data.
Goepfert et al. [38]	PiCCO	RCT	100	PiCCO group had significantly fewer postoperative complications, decreased time to achieve ICU discharge criteria, and decreased length of ICU stay.
